# Different genotype and a liquid whey-supplemented diet influence the resilience of pigs through immune-modulation and anti-inflammatory response

**DOI:** 10.3389/fvets.2022.1046101

**Published:** 2022-11-03

**Authors:** Enrico D'Alessandro, Francesca Arfuso, Viviana Floridia, Giuseppe Tardiolo, Francesco Fazio, Claudia Giannetto, Giuseppe Piccione, Alessandro Zumbo

**Affiliations:** Department of Veterinary Sciences, University of Messina, Messina, Italy

**Keywords:** Nero Siciliano pig, acute-phase proteins, crossbreed, liquid whey supplementation, serum protein electrophoresis

## Abstract

This study evaluated (i) whether weight gain and levels of inflammatory and immune markers including white blood cells (WBC), serum haptoglobin, C-reactive protein, albumin, and globulin fractions change between the Nero Siciliano pig breed and the crossbreed Landrace x Large White (LxLW) reared under the same environmental and farming conditions; and (ii) whether a liquid whey diet supplementation affects the investigated parameters in both genotypes. In this study, 10 crossbreed LxLW and 10 Nero Siciliano pigs were given control feed, representing the control groups (CTRC and CTRNS), whereas 10 crossbreed LxLW and 10 Nero Siciliano pigs were given control feed supplemented with liquid whey for 2 months, representing the experimental groups (WC and WNS). From each pig, body weight and blood were collected before experimental diet supplementation (T0), and one (T1) and two (T2) months after the start of the diet supplemented with whey. The white blood cell count (WBC), serum haptoglobin, C-reactive protein, total proteins, albumin and globulin fraction concentration were assessed. Two-way analysis of variance showed an increasing trend of body weight both in the control and experimental groups of the two pig genotypes throughout the monitoring period (*p* < 0.01) without a significant effect of genotype and diet (*p* > 0.05). The concentration of haptoglobin, β1- and β2-globulins was affected by pig genotype, diet supplementation, and time (*p* < 0.01). The values of WBC, C-reactive protein, albumin, α-globulins, and A/G ratio were affected by diet supplementation (*p* < 0.01) and time (*p* < 0.01) without an influence of genotype (*p* > 0.05). Nero Siciliano pigs showed lower levels of haptoglobin, β1-globulin, and β2-globulin compared to crossbreed LxLW. Nero Siciliano pigs and crossbred LxLW fed with liquid whey showed lower levels of WBC, haptoglobin, C-reactive protein, α-, β1-, and β2-globulins and higher values of albumin compared to control groups. The results reinforced the hypothesis that autochthonous breeds possess higher resilience to farming conditions when compared to allochthonous breeds. Moreover, an immune-modulatory and an anti-inflammatory power of liquid whey dietary supplementation is suggested probably thanks to its content in natural bioactive substances including anti-inflammatory cytokines and anti-oxidative factors.

## Introduction

Reaching high production levels in livestock farms poses important challenges that lead to impairment of animal welfare with consequently increased occurrence of multifactorial diseases and use of veterinarian drugs as well as decreased life expectancy. Various management strategies, including diet, genetic selection, improvement of environmental conditions, and animal health monitoring, should be implemented to secure adequate animal performance without neglecting animal welfare (1, 2). It is well-known that diet is the main factor affecting animal development and growth. Indeed, a nutrient-rich and balanced diet can help to develop and support a healthy and well-functioning immune system (3). It has been suggested that dietary supplementation with liquid whey, a protein-enriched powder, improves some growth, gut health, and immunity parameters in pigs (4, 5). Emerging evidence has demonstrated the usefulness of a whey supplementation diets for the treatment of many gastrointestinal disorders (6, 7). The beneficial effects of liquid whey supplementation have been demonstrated thanks to its numerous bioactive compounds, including functional amino acids, lactoferrin, and growth factors, which stimulate mucin synthesis and modulate the immune response of animals (8, 9). During the last decade, the study of acute phase response (APR) in livestock has aroused growing interest from the scientific community thanks to its value as a disease marker. The APR is defined as an innate, non-specific defense mechanism that is activated in response to infection, inflammation, and immunological disease (10–12). It is a multi-factorial response orchestrated by a combination of cytokines including interleukin-1, interleukin-6, and tumor necrosis factor-α (13). The complex reaction of APR involves the changes in the concentration of some proteins, mainly synthesized in the liver, which are collectively known as acute-phase proteins (APPs). Some of these proteins are called positive APPs as their serum levels increase during the APR, while others are called negative APPs as their serum levels decrease during this response. Both positive and negative APPs have been used in veterinary medicine to study the physiological alterations that take place during the APR to inflammation and infection (10–12). However, the behavior of APPs, intended as the type and the intensity of the concentration change, differs between species (12). Several investigations have indicated that the assessment of the serum APP concentrations in pigs will provide not only useful diagnostic information on the health of individual animals (14, 15) but also a basis to improve herd health (16, 17). Furthermore, it has been proposed that the measurement of APPs in blood samples of pigs could be of practical use at slaughterhouses for the detection of sick animals during the meat inspection process (12, 18–20). The APP assay has also been shown to be an interesting tool for the evaluation of antibiotic treatment efficacy (21, 22). It has been proposed that autochthonous, more rustic breeds show higher resistance to inflammation and higher resilience (23, 24). Moreover, the production system of the local breed has a lower environmental impact compared to the intensive type (25). Among Italian autochthonous pig breeds, the Nero Siciliano pig is the most famous thanks to its high-quality meat products. Although the Nero Siciliano pigs showed a lower growth performance, being not competitive for production traits compared to allochthonous pig breeds, they appear to withstand adverse climatic conditions and resist disease (25, 26). Efforts to improve the knowledge of genetic and phenotypic traits of local breeds, intended as sources of genomic diversity for the improvement of pigs for commercial use, are constantly growing (27, 28). However, information relating to the immune and inflammatory status of Nero Siciliano pigs is not available in the scientific literature, so far. Because of such consideration, this study (i) evaluated whether the levels of the main inflammatory and immune markers including serum haptoglobin, C-reactive protein, white blood cells, albumin, and globulin fractions change between the Nero Siciliano pigs and the crossbreed pigs reared under the same environmental and farming conditions; and (ii) to assess whether diet supplementation with liquid whey affects the levels of considered inflammatory and immune markers in both pig genotypes.

## Materials and methods

### Animal management

The study was approved by the Animal Experiment Ethics Committee of Messina University (Authorization number 055_2021) according to the European guidelines for the care and use of animals in research (Directive 2010/63/EU 2010). All procedures were carried out according to relevant guidelines and regulations. The trial involved 40 female pigs: 20 crossbred LxLW (Landrace × Large White), and 20 Nero Siciliano pigs homogeneous for body weight (average initial body weight of 19.4 ± 1.92 kg), age (58 ± 2 days), and breeding management. All pigs were kept in individual pens with nipple waterers and fed individually. The animals were divided into four groups (i.e., 10 individuals each), with two control groups (crossbreed LxLW, CTRC; Nero Siciliano pigs, CTRNS) fed with a pellet complete feed (Table 1) rationed based on 3% of the live weight, and two treatment groups (treatment crossbred LxLW, WC; treatment Nero Siciliano pigs, and WNS) receiving the same diet integrated with fresh liquid whey (5% carbohydrate, 0.8% proteins, 0.6% fat, 93% water, and 0.6% ash) at the level of 1.5 L/day/pig for 8 weeks. Liquid whey was fed separately from the formula feed, using a wet feeder. Pens were provided with nipple waterers and stainless-steel feeders. Animals had no gastrointestinal diseases or any antibiotic exposure prior to the study. The trial lasted for a total of 60 days, from day 58 until day 118 of life, including an adaptation period of 15 days. All animals had no access to the outside and they were exposed to a natural photoperiod and natural environmental temperature (Table 2). Thermal and hygrometric records were carried out inside and outside the pen for the whole study by means of a data logger (Gemini, UK).

**Table 1:** Ingredients and composition of the pelleted complete feed.

**Ingredients**	**g/kg of Dry matter**
Corn	550
Broad bean	125
Peas bean	110
Sunflower meal (38% crude protein)	80
Wheat middling	70
Carob	30
Sugar cane molasses	13
**Analytical components**	% on a wet basis
Crude protein	17.4
Crude fat	5.7
Crude fiber	4.5
Ash	5.3
Calcium	0.6
Phosphorus	0.5
Sodium	0.2
Lysine	1.2
Methionine	0.4
**Additive components**	
Vitamin B1	1.0 mg
Vitamin B2	3.0 mg
Vitamin B6	1.5 mg
Vitamin B12	0.015 mg
Vitamin D3	(1,000 UI)
Vitamin E	20 mg
Vitamin K3	1.0 mg
Niacin	15.0 mg
Calcium-D	10.3 mg
Choline	200 mg
Cu	14.0 mg
Fe	89.8 mg
I	0.50 mg
Mn	39.9 mg
Se	0.15 mg
Zn	99.6 mg
Biotine	0.10 mg
DL-Methionine	0.12 mg
Lysine	500 mg

Data were provided by the feed manufacturing industry.

**Table 2:** Environmental parameters measured inside and outside the pen before (May, T0), and one (June, T1) and two (July, T2) months after the starting of experimental diet supplemented with whey.

	**Experimental period**					
**Environmental parameters**	**T0**		**T1**		**T2**	
	**Inside**	**Outside**	**Inside**	**Outside**	**Inside**	**Outside**
Temperature (°C)	21.5	22	24.5	25	26	26.5
Relative humidity (%)	61	62	61	62	53	55

### Sampling procedures and laboratory analysis

From each crossbreed white pig and Nero Siciliano pig belonging to control groups (CTRC and CTRNS, respectively) and crossbreed white pig and Nero Siciliano pig belonging to experimental groups (WC and WNS, respectively), body weight measurement and blood sampling were performed before experimental diet supplementation (T0), and one (T1) and two (T2) months after the start of the diet supplemented with whey. Blood samples were collected from each pig by jugular venipuncture into vacutainer tubes containing EDTA and into tubes with clot activator (Terumo Corporation Japan). After collection, all blood samples were immediately cooled in an ice-water bath. Blood samples collected into EDTA tubes were analyzed within 2 h after collection by means of an automated hematology analyzer (HeCo Vet C; SEAC, Florence, Italy) in order to assess white blood cell count (WBC); whereas, the tubes without anticoagulant agent were centrifuged at 1,900 × *g* for 16 min at 4°C in order to obtain serum that was aliquoted and stored at −20°C until analysis. On obtained serum samples, the concentration of haptoglobin, C-reactive protein, total proteins, albumin, and globulin fractions were determined. Specifically, the concentration of haptoglobin and C-reactive protein was assessed using ELISA kits specific for pig species (Haptoglobin kit, PHASETM RANGE, Tridelta Ltd, Maynooth, Ireland, Sensitivity 0.005 mg/ml; the intra- and the inter-assay coefficients of variation <7 and <6%, respectively; Porcine C-Reactive Protein kit, PHASE RANGE, Tridelta Ltd, Maynooth, Ireland, Sensitivity 0.001 mg/ml; the intra- and the inter-assay coefficients of variation <5 and <4%, respectively) by means of a microtiter plate reader (EZ Read 400 ELISA, Biochrom, Cambridge, United Kingdom). All calibrators and samples were run in duplicate, and samples exhibited parallel displacement to the standard curve for both ELISA analyses. Serum total protein concentration was assessed with a commercially available kit by means of an automated ultraviolet (UV) spectrophotometer (Slim; SEAC, Florence, Italy) using the biuret method and bovine albumin at a concentration of 6.02 g/dL as a standard (Biosystems S.A., Barcelona, Spain). Electrophoresis for protein fraction assessment was performed using an automated system (Selvet24, Seleo Engineering, Naples, Italy) according to the procedures described by the manufacturer. A total of 25 μl of each serum sample was applied to numbered sample wells of acetate cellulose films. Each holder accommodated up to 24 samples. Films were electrophoresed for 28 min at 180 V. After electrophoresis, films were simultaneously fixed using an automated system, stained in red stain acid solution for 10 min, and then dried at 37°C. After destaining in acetic acid and drying completely for 15 min films were scanned on a densitometer and electrophoretic curves plus related quantitative specific protein concentrations for each sample were displayed, using computer software (SelVet 24). All samples were analyzed by the same operator, who determined the lines separating fractions in the densimeter tracing. The major protein fractions were divided into albumin, α-, β1-, β2-, and γ-globulins, from the cathode to the anode, according to the recommendation by the manufacturer and with previous findings on pig species (29). Relative protein concentrations within each fraction were determined as the optical absorbance percentage; then the absolute concentration (g/dL) and albumin/globulin ratio (A/G) were calculated using the total protein concentration.

### Statistical analysis

The normal distribution of data was verified by the application of the Shapiro-Wilk test. The results were normally distributed (*p* > 0.05) and were subjected to two-way repeated measures analysis of variance (ANOVA) in order to assess the significant effect of genotype and whey supplementation. Bonferroni multiple comparison test was applied for *post-hoc* mean comparison. Significance was declared at *p* < 0.05. The data were analyzed using the software Prism v. 9.00 (Graphpad Software Ltd., San Diego, CA, USA, 2020).

## Results

**Figure 1:**
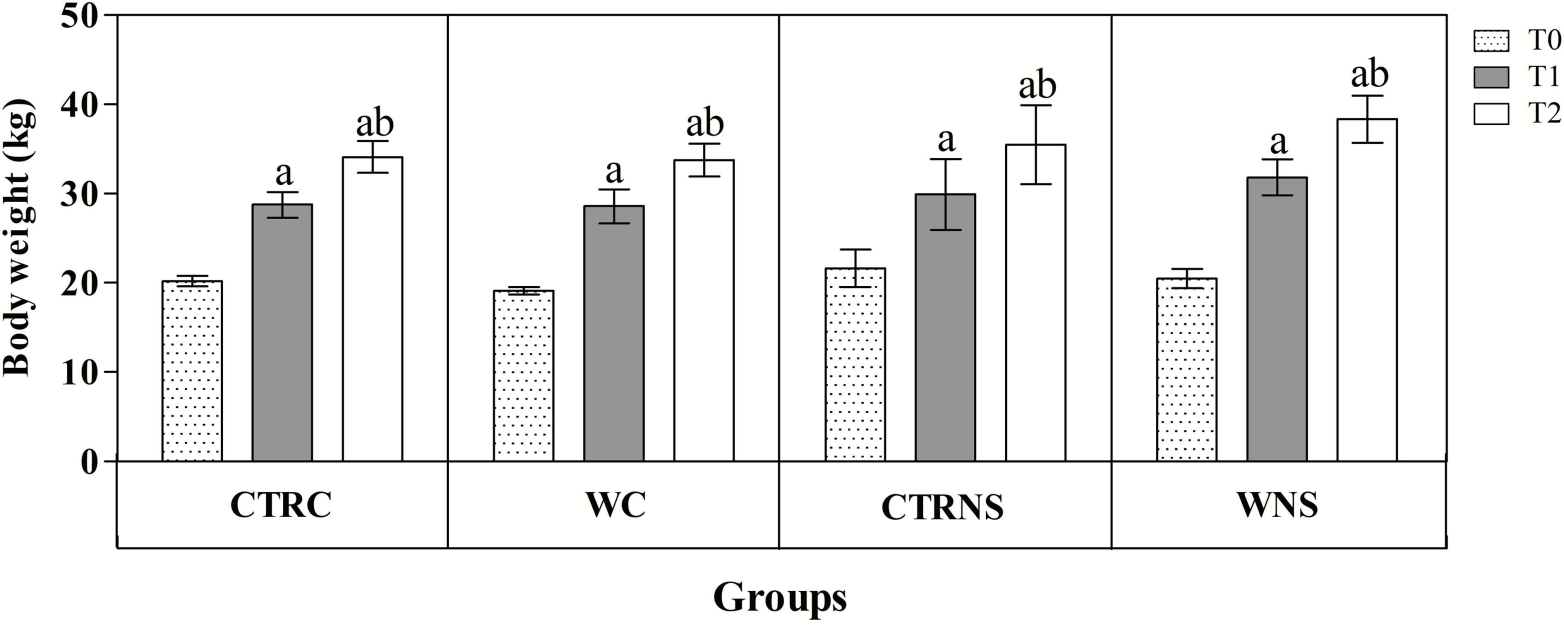
Mean values ± standard error of the mean (±SEM) of body weight recorded from pigs belonging to control fed and experimental dietary supplemented crossbreed LxLW groups (CTRC and WC) and Nero Siciliano breed (CTRNS and WNS) before (T0), one (T1) and two (T2) months after the starting of diet supplemented with whey.

**Figure 2:**
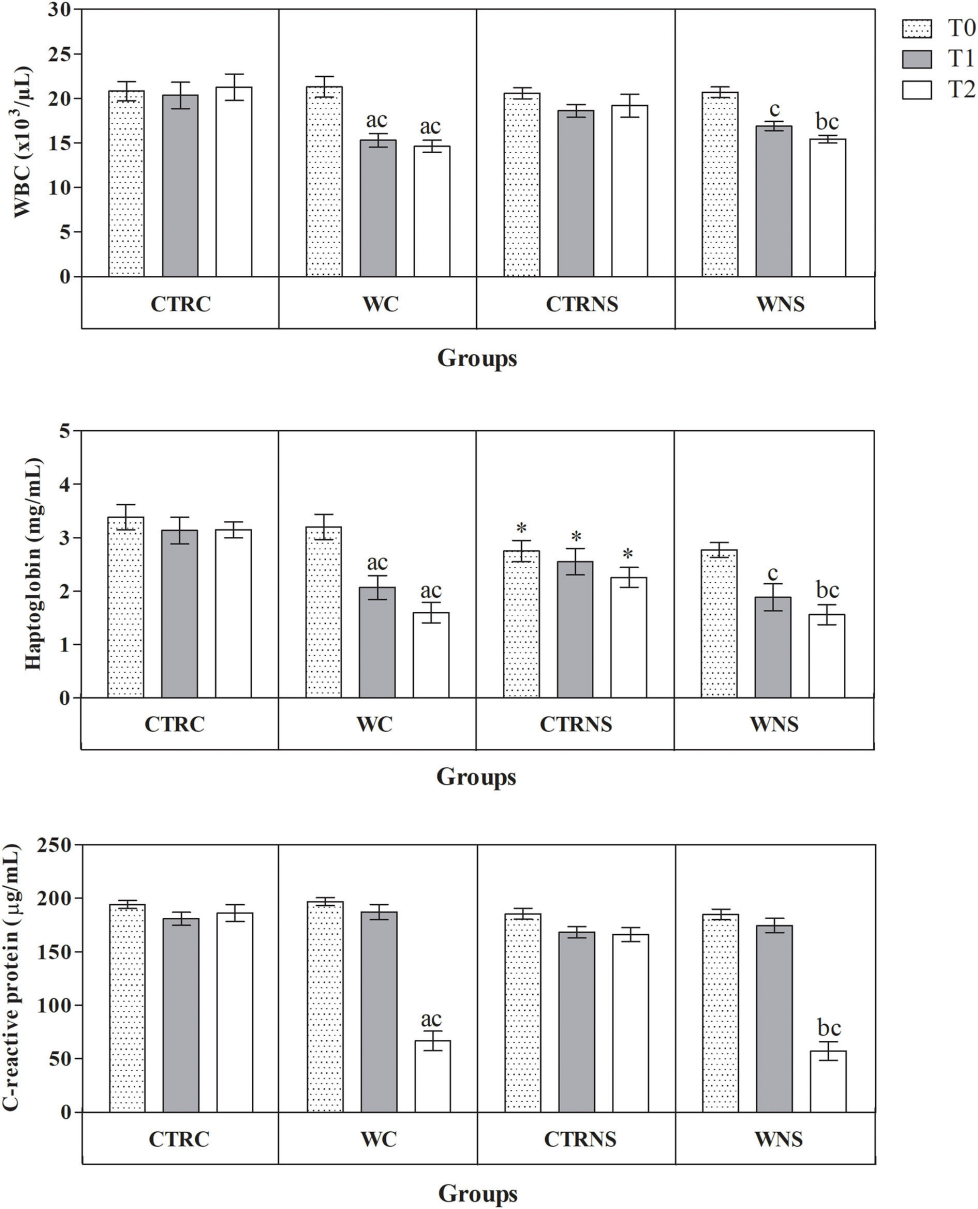
Mean values ± standard error of the mean (±SEM) of white blood cell count (WBC), serum haptoglobin and C-reactive protein obtained from pigs belonging to control fed and experimental dietary supplemented crossbreed LxLW groups (CTRC and WC) and Nero Siciliano breed (CTRNS and WNS) before (T0), one (T1) and two (T2) months after the starting of diet supplemented with whey.

**Figure 3:**
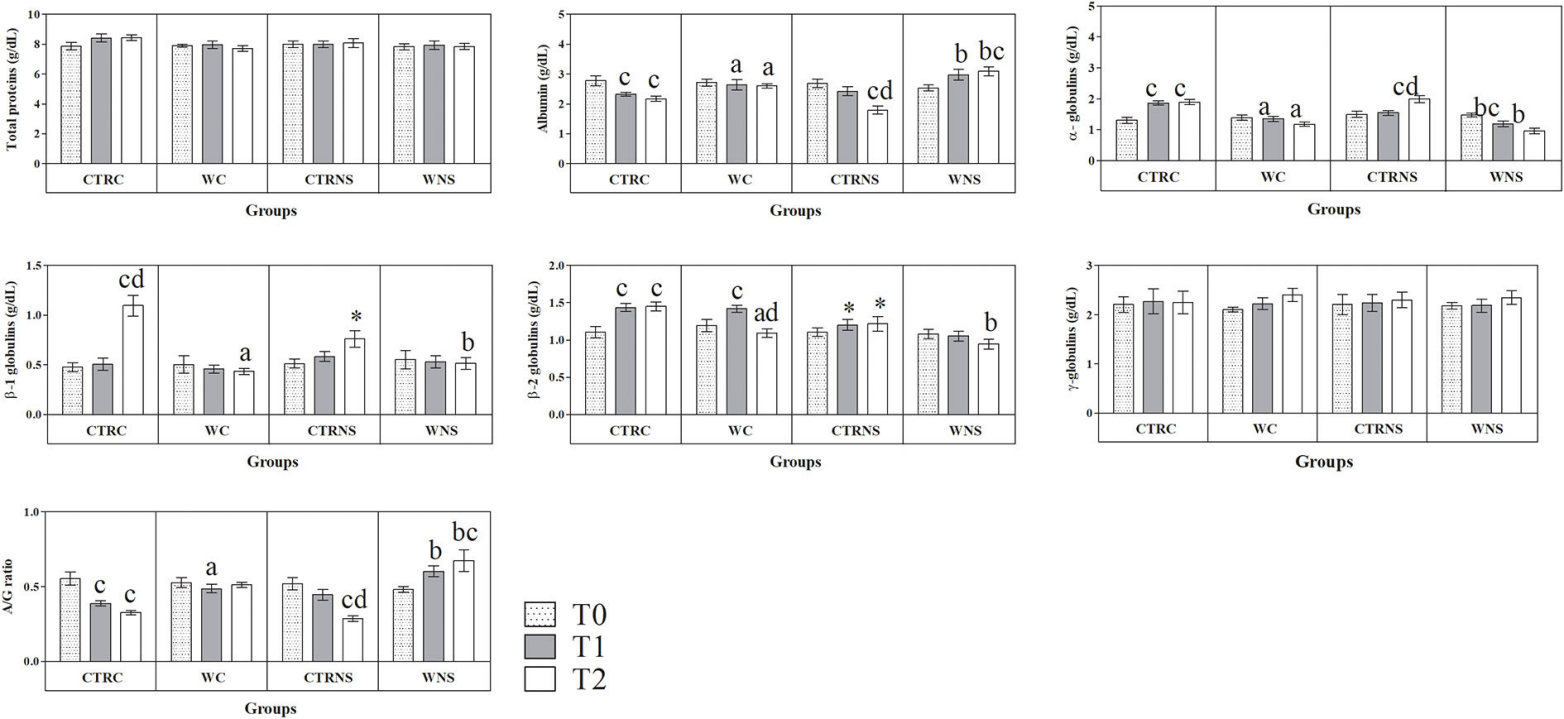
Mean values ± standard error of the mean (±SEM) of serum total proteins, albumin, α-, β1-, β2- and γ-globulins, and A/G ratio obtained from pigs belonging to control fed and experimental dietary supplemented crossbreed LxLW groups (CTRC and WC) and Nero Siciliano breed (CTRNS and WNS) before (T0), one (T1) and two (T2) months after the starting of diet supplemented with whey.

All the results are expressed as the mean ± standard error of the mean (SEM). As shown in Figure 1, the values of body weight showed a significant increasing trend throughout the monitoring period both in the control and experimental groups of the two pig genotypes (*p* < 0.01) without a significant effect of genotype and diet (*p* > 0.05). The average daily weight recorded was 308 g/head per day in pigs of CTRC, 305 g/head per day in pigs of CTRNS, 315 g/head per day in pigs of WC, and 310 g/head per day in pigs of WNS. The feed conversion rate recorded was 2.78 kg/kg in pigs of CTRC, 2.80 kg/kg in pigs of CTRNS, 2.74 kg/kg in pigs of WC, and 2.77 kg/kg in pigs of WNS. The serum concentration of haptoglobin, β1-, and β2-globulins was affected by pig genotype and experimental diet supplementation (*p* < 0.01, Figures 2, 3). Contrariwise, the values of C-reactive protein, WBC (Figure 2), albumin, α-globulins, and A/G ratio (Figure 3) were affected by diet supplementation (*p* < 0.01) without an influence of genotype (*p* > 0.05). Statistical analysis of the data showed no significant difference related to the pig's genotype and diet supplementation in the values of serum total proteins and γ-globulins (*p* > 0.05, Figure 3). Control Nero Siciliano pigs (CTRNS) showed lower values of haptoglobin at T0, T1, and T2, β1-globulins at T2, and β2-globulins at T1 and T2, than control crossbreed LxLW (CTRC). Both pig groups fed a diet supplemented with liquid whey (WC and WNS) showed lower values of haptoglobin, C-reactive protein, WBC, α-, β1-, and β2-globulins, and higher values of albumin and A/G ratio compared to control groups (CTRC and CTRNS) (*p* < 0.01). Regarding the time-related changes of investigated parameters, a significant decreasing trend (*p* < 0.01) of haptoglobin, C-reactive protein, and WBC was found from T0 up to T2 in WC and WNS; albumin, and A/G ratio values decreased at T1 and T2 respect to T0n CTRC and CTRNS, while increased at T2 compared to T0 and T1 in WNS (*p* < 0.01); α-globulins showed increased values (*p* < 0.01) at T1 and T2 than T0 in CTRC and CTRNS, and decreased values at T2 than T0 in WNS (*p* < 0.01); β1-globulins showed higher values at T2 than T0 and T1 in CTRC (*p* < 0.01), whereas β2-globulins showed higher values at T1 and T2 than T0 in CTRC (*p* < 0.01), and higher values at T1 than T0 and T2 in WNS (*p* < 0.01).

## Discussion

Despite the increase in interest in local breeds and their production systems thanks to their high-quality products, compared with industrial genotypes, scant data on the physiological responses of autochthonous pig breeds is currently available. To the best of the authors' knowledge, this study represents the first study assessing the concentration of the main markers of immune and inflammatory response in the autochthonous Nero Siciliano pig breed and the influence of a 2-month liquid whey supplementation diet on the weight gain and the levels of considered immune and inflammatory markers in this local pig breed. The concentrations of all investigated parameters fall within the reference ranges suggested for pig species (29, 30); however, a genotype-related effect was observed on the values of haptoglobin, β1-, and β2-globulins. According to this study, the Nero Siciliano pigs of both the control and experimental groups showed lower levels of haptoglobin, β1-, and β2-globulins compared to allochthonous crossbreed pig groups. Contrariwise, the values of body weight, WBC, C-reactive protein, total proteins, albumin, α- and γ-globulins, and A/G ratio were comparable between Nero Siciliano and crossbreed LxLW pigs. During the 2 months of monitoring, a dynamic change in the investigated parameters was observed in the two pig genotypes. A decreasing trend of albumin and an increasing trend of α-globulin values were observed in control groups of Nero Siciliano and crossbred LxLW pigs throughout the monitoring period. The values of β1- and β2-globulins remained unchanged in Nero Siciliano pigs throughout the study, whereas they progressively increased in crossbred LxLW pigs belonging to the control group. In pigs, haptoglobin is likely to be the best APPs marker of inflammation (13, 31, 32). As a matter of fact, the haptoglobin concentration was higher in pigs under the effect of stressors such as road transport or changes in the pattern of food administration compared to control animals (33, 34); furthermore, increased levels of haptoglobin have been found to be correlated with decreased weight gain (33, 35). It is well-known that the changes in β1-globulins, including C3 and C4 complement, transferrin, and ferritin, and the β2-globulins, including fibrinogen, are involved in the acute-phase response, as well. It has been suggested that more severe inflammation is linked to the impairment of immune system and to a reduction in performance in livestock (23, 24, 36–40). Overall, the results suggested that, though an activation of acute-phase response to farming conditions happened in autochthonous and allochthonous pig genotypes, the Nero Siciliano pigs showed an attenuated inflammatory status, highlighted by lower haptoglobin, β1-, and β2-globulins levels, than crossbred LxLW pigs. Therefore, it strengthens the hypothesis, widely shared by the scientific community, that autochthonous breeds possess higher resilience to farming conditions and higher resistance to disease when compared to allochthonous breeds (23, 24). The Nero Siciliano pig breed appears to withstand adverse climatic conditions and resist disease (25). This breed is characterized by an evident polymorphism influenced by the natural living environment, the rearing systems, and the type of targeted production system (25). It has been suggested that allochthonous breeds are more susceptible to common environmental stressors in terms of housing, hygiene, and feeding conditions, with crucial repercussions on disease occurrence (23, 41). A progressive increase in weight values was observed in both control and experimental pigs of each breed throughout the monitoring period, though no genotype- and diet-related differences were observed. The average daily weight and the feed conversion rate obtained for pigs of each group fall within the normal values referred to by the farm breeding system. These findings suggest that the liquid whey supplementation did not negatively affect the weight of pigs in the fattening stage of their growth, confirming the good health status of the animals. The whey supplementation diet influenced most of the parameters investigated in both Nero Siciliano and crossbred LxLW pigs. Specifically, the values of WBC, haptoglobin, C-reactive protein, α-, β1-, and β2-globulins were lower in Nero Siciliano and crossbreed LxLW pigs fed liquid whey dietary supplement compared to control groups. Contrariwise, albumin, a negative APP, showed higher values in experimental diet supplementation groups compared to control ones. Noteworthy, in both Nero Siciliano and crossbred LxLW pigs, liquid whey dietary supplement showed a significant effect on WBC, haptoglobin, albumin and α-globulins concentration starting from 1 month after its administration, whereas the whey dietary supplementation influenced the values of C-reactive protein, β1-, and β2-globulins 2 months after its administration. The faster response of haptoglobin to the experimental diet compared to C-reactive protein was expected. Indeed, though these APPs are likely to be the best markers of inflammation, the haptoglobin response is known to be faster than that of C-reactive protein in pigs (13). The serum concentration of haptoglobin is considered an index of subclinical illness and, therefore, of the animal health status in pigs (42). According to previous studies carried out on swine (4, 5), the results herein obtained seem to highlight the immune-modulatory and anti-inflammatory power of whey dietary supplementation. The whey supplementation contains not only essential nutrients but also a number of natural bioactive substances, including anti-inflammatory cytokines and anti-oxidative factors (e.g., transforming growth factor-β1, lactoferrin, α-lactalbumin, lactoperoxidase, and lysozyme) that have many beneficial effects on animal health (5, 43). It has been well-established that whey protein concentrate improves intestinal barrier function protecting against the interferon-γ-induced barrier impairment (9). Supplementation with 5% whey improved intestinal morphology by increasing jejunal villus height and villus height:crypt depth ratio in lipopolysaccharide (LPS)-induced intestinal injury damage (4, 44). It has been shown that whey dietary supplementation reduced the expression of the TNF-α, IL-1β, IL-6, and IL-8 genes compared with the LPS-challenged pigs (4). Furthermore, whey product has demonstrated a number of anti-inflammatory effects, including decreased cytokine release in rodent models after exposure to LPS (45).

## Conclusion

In order to enhance the local breed's sustainability, it is of paramount importance to get a scientific assessment of their growth performance, immune and inflammatory responses under specific environmental and farming conditions. In this study, the values of the main markers of immune and inflammatory response and the possible influence of a 2-month whey supplementation diet were assessed in the autochthonous Nero Siciliano pig breed and in the crossbred LxLW. The results gathered in the present study showed a genotype-related influence was observed on the values of haptoglobin, β1-, and β2-globulins. In particular, the Nero Siciliano pigs showed lower levels of haptoglobin, β1-, and β2-globulins compared to the crossbred LxLW. Furthermore, the whey-supplemented diet affected the main markers of the immune and inflammatory status of pigs. In this regard, the Nero Siciliano and the corssbred LxLW fed liquid whey dietary supplement showed lower levels of WBC, haptoglobin, C-reactive protein, α-, β1-, and β2-globulins and higher values of albumin compared to control groups. The current study reinforces the hypothesis, widely shared by the scientific community, that autochthonous breeds possess higher resilience to farming conditions and higher resistance to disease when compared to allochthonous breeds and/or crossbreeds. Furthermore, it could be suggested an immune-modulatory and an anti-inflammatory power of whey dietary supplementation thanks to its content in natural bioactive substances including anti-inflammatory cytokines and anti-oxidants. Further studies on a greater number of animals are needed in order to better characterize the immune and inflammatory status of Nero Siciliano pigs in order to adopt protocols for early and predictive diagnosis of production diseases to minimize drug usage in farms. A deep knowledge of the physiological response of this Sicilian autochthonous breed to housing and feeding conditions throughout its breeding is required to assess animal health status and optimize animal welfare.

## Data availability statement

The raw data supporting the conclusions of this article will be made available by the authors, without undue reservation.

## Ethics statement

The animal study was reviewed and approved by Animal Experiment Ethics Committee of Messina University (Authorization number 055_2021). Written informed consent was obtained from the owners for the participation of their animals in this study.

## Author contributions

Conceptualization, data curation, and project administration: ED and AZ. Methodology: VF, FA, GP, GT, and CG. Software: FA and ED. Validation: AZ and GP. Investigation: ED, FF, CG, GT, and GP. Writing-original draft preparation and formal analysis: FA. Writing-review and editing: FA, ED, and GP. Supervision: ED, GP, and AZ.

## Funding

This research was supported by a grant from a FEASRPSR Sicily 2014-2020, Misura 10, Sottomisura 10.2 b, CUP G49J21003940009.

## Conflict of interest

The authors declare that the research was conducted in the absence of any commercial or financial relationships that could be construed as a potential conflict of interest.

## Publisher's note

All claims expressed in this article are solely those of the authors and do not necessarily represent those of their affiliated organizations, or those of the publisher, the editors and the reviewers. Any product that may be evaluated in this article, or claim that may be made by its manufacturer, is not guaranteed or endorsed by the publisher.

## References

[1] Stein HH. Experience of feeding pigs without antibiotics: a European perspective. Anim Biotech. (2002) 13:85–95. 10.1081/ABIO-120005772 12212947

[2] Stein HH Kil DY. Reduced use of antibiotic growth promoters in diets fed to weanling pigs: dietary tools, part 2. Anim Biotechnol. (2006) 7:217–31. 10.1080/10495390600957191 17127532

[3] Klasing KC Leshchinsky TV. Interactions between nutrition and immunity. In: Gershwin ME German JB Keen CL, editors. Nutrition and Immunology. Totowa, NJ: Humana Press (2000).

[4] Xiao K Jiao L Cao S Song Z Hu C Han X. Whey protein concentrate enhances intestinal integrity and influences transforming growth factor-β1 and mitogen-activated protein kinase signalling pathways in piglets after lipopolysaccharide challenge. Br J Nutr. (2016) 115:984–93. 10.1017/S0007114515005085 26810899

[5] Nielsen CH Hui Y Nguyen DN Ahnfeldt AM Burrin DG Hartmann B. Alpha-Lactalbumin enriched whey protein concentrate to improve gut, immunity and brain development in preterm pigs. Nutrients. (2020) 12:245. 10.3390/nu12010245 31963562 PMC7020014

[6] Sprong RC Schonewille AJ van der Meer R. Dietary cheese whey protein protects rats against mild dextran sulfate sodium-induced colitis: role of mucin and microbiota. J Dairy Sci. (2010) 93:1364–71. 10.3168/jds.2009-2397 20338413

[7] Playford RJ Macdonald CE Johnson WS. Colostrum and milk-derived peptide growth factors for the treatment of gastrointestinal disorders. Am J Clin Nutr. (2000) 72:5–14. 10.1093/ajcn/72.1.5 10871554

[8] Marshall K. Therapeutic applications of whey protein. Altern Med Rev. (2004) 9:136–56. 15253675

[9] Hering NA Andres S Fromm A van Tol EA Amasheh M Mankertz J. Transforming growth factor-β, a whey protein component, strengthens the intestinal barrier by upregulating claudin-4 in HT-29/B6 cells. J Nutr. (2011) 141:783–89. 10.3945/jn.110.137588 21430244

[10] Baumann H Gauldie J. The acute phase response. Immunol Today. (1994) 15:74–80. 10.1016/0167-5699(94)90137-6 7512342

[11] Gruys E Obwolo MJ Toussaint MJM. Diagnostic significance of the major acute phase proteins in veterinary clinical chemistry: a review. Vet Bull. (1994) 64:1009–18.

[12] Petersen HH Nielsen JP Heegard PMH. Application of acute phase protein measurements in veterinary clinical chemistry. Vet Res. (2004) 35:163–87. 10.1051/vetres:2004002 15099494

[13] Eckersall PD Saini PK McComb C. The acute phase response of acid soluble glycoprotein, alpha(1)-acid glycoprotein, ceruloplasmin, haptoglobin and C-reactive protein, in the pig. Vet Immunol Immunopathol. (1996) 51:377–85. 10.1016/0165-2427(95)05527-4 8792574

[14] Itoh H Tamura K Izumi M Motoi Y Kidoguchi K Funayama Y. The influence of age and health-status on the serum alpha1 -acid glycoprotein level of conventional and specific pathogen-free pigs. Can J Vet Res. (1992) 57:74–8. PMC1263597 8490810

[15] Hall WF Eurell TE Hansen RD Herr LC. Serum haptoglobin concentration in swine naturally or experimentally infected with actinobacillus-pleuropneumoniae. J Amer Vet Med Assoc. (1992) 201:1730–33. 1293115

[16] Segalés J Piñeiro C Lampreave F Nofrarías M Mateu E Calsamiglia M. Haptoglobin and pig-major acute protein are increased in pigs with postweaning multisystemic wasting syndrome (PMWS). Vet Res. (2004) 35:275–82. 10.1051/vetres:2004009 15210076

[17] Burger W Fennert EM Pohle M Wesemeier H. C-reactive protein - a characteristic feature of health control in swine. J Vet Med Ser A. (1992) 39:635–38. 10.1111/j.1439-0442.1992.tb00227.x 1455931

[18] Skinner JG. International standardization of acute phase proteins. Vet Clin Pathol. (2001) 30:2–7. 10.1111/j.1939-165X.2001.tb00248.x 12024323

[19] Saini PK Webert DW. Application of acute phase reactants during antemortem and postmortem meat inspection. J Am Vet Med Ass. (1991) 198:1898–901. 1714890

[20] Yamane H Kanouchi H Arimizu G Obi T Oka T. Increases in pig major acute-phase protein in wasting pigs brought to the abattoir. J Vet Med Sci. (2006) 68:511–13. 10.1292/jvms.68.511 16757898

[21] Lauritzen B Lykkesfeldt J Skaanild MT Angen O Nielsen JP Friis C. Putative biomarkers for evaluating antibiotic treatment: an experimental model of porcine Actinobacillus pleuropneumoniae infection. Res Vet Sci. (2003) 74:261–70. 10.1016/S0034-5288(03)00028-6 12726745

[22] Hulten C Johansson E Fossum C Wallgren P. Interleukin 6, serum amyloid A and haptoglobin as markers of treatment efficacy in pigs experimentally infected with Actinobacillus pleuropneumoniae. Vet Microbiol. (2003) 95:75–89. 10.1016/S0378-1135(03)00136-6 12860078

[23] Curone G Filipe J Cremonesi P Trevisi E Amadori M Pollera C. What we have lost: Mastitis resistance in Holstein Friesians and in a local cattle breed. Res Vet Sci. (2018) 116:88–98. 10.1016/j.rvsc.2017.11.020 29223308

[24] Martins JM Fialho R Albuquerque A Neves J Freitas A Nunes JT. Growth, blood, carcass and meat quality traits from local pig breeds and their crosses. Animal. (2020) 14:636–47. 10.1017/S1751731119002222 31578161

[25] Zumbo A Sutera AM Tardiolo G D'Alessandro E. Sicilian Black Pig: an overview. Animals. (2020) 10:2326. 10.3390/ani10122326 33297476 PMC7762396

[26] D'Alessandro E Giosa D Sapienza I Giuffrè L Aiese CR Romeo O. Whole genome SNPs discovery in Nero Siciliano pig. Genet Mol Biol. (2019) 42:594–602. 10.1590/1678-4685-gmb-2018-0169 31188930 PMC6905442

[27] Chen C D'Alessandro E Murani E Zheng Y Giosa D Yang NS. SINE jumping contributes to large-scale polymorphisms in the pig genomes. Mob DNA. (2021) 12:17. 10.1186/s13100-021-00246-y 34183049 PMC8240389

[28] D'Alessandro E Sottile G Sardina MT Criscione A Bordonaro S Sutera AM. Genome-wide analyses reveal the regions involved in the phenotypic diversity in Sicilian pigs. Anim Genet. (2020) 51:101–5. 10.1111/age.12887 31793034

[29] Kaneko JJ. Clinical Biochemistry of Domestic Animals. Berkeley, CA: Academic Press (1989). p. 142–65.

[30] Weiss DJ Wardrop KJ. Schalm's Veterinary Hematology. Philadelphia, PA: Lippincott Williams and Wilkins (2010).

[31] Knura-Deszczka S Lipperheide C Petersen B Jobert JL Berthelot-Herault F Kobisch M. Plasma haptoglobin concentration in swine after challenge with Streptococcus suis. J Vet Med B. (2002) 49:240–44. 10.1046/j.1439-0450.2002.00556.x 12121045

[32] Carpintero R Piñeiro M Andrés M Iturralde M Alava MA Heegaard PM. The concentration of apolipoprotein A-I decreases during experimentally induced acute-phase processes in pigs. Infect Immun. (2005) 73:3184–7. 10.1128/IAI.73.5.3184-3187.2005 15845530 PMC1087351

[33] Piñeiro C Piñeiro M Morales J Carpintero R Campbell FM Eckersall PD. Pig acute-phase protein levels after stress induced by changes in the pattern of food administration. Animal. (2007) 1:133–9. 10.1017/S1751731107283909 22444216

[34] Piñeiro M Piñeiro C Carpintero R Morales J Campbell FM Eckersall PD. Characterisation of the pig acute phase protein response to road transport. Vet J. (2007) 173:669–74. 10.1016/j.tvjl.2006.02.006 16584904

[35] Clapperton M Bishop SC Cameron ND Glass EJ. Association of acute phase protein levels with growth performance and with selection for growth performance in Large White pigs. Anim Sci. (2005) 81:213–20. 10.1079/ASC50180213

[36] Trevisi E Moscati L Amadori M. Disease-predicting and prognostic potential of innate immune responses to noninfectious stressors: human and animal models. In: Amadori M, editor. The Innate Immune Response to Non-Infectious Stressors. Netherland: Elsevier (2016). p. 209–35.

[37] Trevisi E Zecconi A Bertoni G Piccinini R. Blood and milk immune and inflammatory profiles in periparturient dairy cows showing a different liver activity index. J Dairy Res. (2010) 77:310–17. 10.1017/S0022029910000178 20576169

[38] Gianesella M Fiore E Arfuso F Vecchio D Curone G Morgante M. Serum haptoglobin and protein electrophoretic fraction modifications in buffaloes (*Bubalus bubalis*) around calving and during early lactation. J Dairy Res. (2019) 86:291–5. 10.1017/S0022029919000438 31292012

[39] Fiore E Barberio A Morgante M Rizzo M Giudice E Piccione G. Glucose infusion response to some biochemical parameters in dairy cows during the transition period. Anim Sci Pap Rep. (2015) 33:129–36. 10.7482/0003-9438-57-003

[40] Armato L Gianesella M Fiore E Rizzo M Giudice E Piccione G. Rumen volatile fatty acid x dietary supplementation with live yeast and yeast cell wall in feedlot beef cattle. Acta Agric Scand A. (2016) 66:119–24. 10.1080/09064702.2016.1272628

[41] Bach A. Associations between several aspects of heifer development and dairy cow survivability to second lactation. J Dairy Sci. (2011) 94:1052–57. 10.3168/jds.2010-3633 21257075

[42] Eurell TE Bane DP Hall WF Schaeffer DJ. Serum haptoglobin concentration as an indicator of weight gain in pigs. Can J Vet Res. (1992) 56:6–9. 1586895 PMC1263495

[43] Sangild PT. Gut responses to enteral nutrition in preterm infants and animals. Exp Biol Med. (2006) 231:1695–711. 10.1177/153537020623101106 17138756

[44] Li Y Østergaard MV Jiang P Chatterton DE Thymann T Kvistgaard AS. Whey protein processing influences formula-induced gut maturation in preterm pigs. J Nutr. (2013) 143:1934–42. 10.3945/jn.113.182931 24047702

[45] Beaulieu J Girard D Dupont C Lemieux P. Inhibition of neutrophil infiltration by a malleable protein matrix of lactic acid bacteria-fermented whey proteins *in vivo*. Inflamm Res. (2009) 58:133–8. 10.1007/s00011-009-7100-y 19184347

